# In search of new anticancer drugs: Data for cytotoxic activities of green synthesized silver nanoparticles from ethanolic extracts of fruits and leaves of *Annona muricata* and 5-Fluorouracil against HeLa, PC3 and PNT1A cell lines

**DOI:** 10.1016/j.dib.2019.104442

**Published:** 2019-08-28

**Authors:** Yahaya Gavamukulya, Esther N. Maina, Amos M. Meroka, Hany A. El-Shemy, Gabriel Magoma, Fred Wamunyokoli

**Affiliations:** a Department of Molecular Biology and Biotechnology, Pan African University Institute for Basic Sciences, Technology and Innovation (PAUSTI), P. O. Box, 62000-00200 Nairobi, Kenya; b Department of Biochemistry and Molecular Biology, Faculty of Health Sciences, Busitema University, P.O. Box, 1460 Mbale, Uganda; c Department of Biochemistry, College of Health Sciences, University of Nairobi, P.O. Box 30197-00100 Nairobi, Kenya; d Department of Biochemistry, School of Medicine and Health Sciences, Kenya Methodist University, P.O. Box 267-60200 Meru, Kenya; e Department of Biochemistry, Faculty of Agriculture, Cairo University, 12613 Giza, Egypt; f Department of Biochemistry, College of Health Sciences, Jomo Kenyatta University of Agriculture and Technology, P. O. Box, 62000-00200 Nairobi, Kenya

**Keywords:** Silver nanoparticles (AgNPs), 5FU, HeLa, PC3, PNT1A, Cytotoxicity, Resazurin

## Abstract

In this article, we present data on the anticancer activities of green synthesized silver nanoparticles (AgNPs) from ethanolic extracts of fruits (AgNPs-F) and leaves (AgNPs-L) of *Annona muricata* and standard anticancer drug 5-Fluorouracil (5-FU) on two cancer cell lines, i.e. cervical adenocarcinoma (HeLa cells) and prostate adenocarcinoma (PC3 cells) as well as on an immortalized normal prostate cell line, PNT1A. The cytotoxicity on the cells was determined by measuring the absorbance signal of resazurin dye. It has long been known that metabolically active cells change the resazurin from blue (oxidized) to red (reduced) forms, corresponding to the absorbance signals at a wavelength of 570nm (A570) and 600nm (A600) respectively, from which therefore the effects of any treatments on percentage cell viability/death can be elucidated. The raw data values of the treatments against the HeLa, PC3 and PNT1A cells are shown in the different Tables. Examples of how the data can be analyzed have been illustrated using different growth inhibition curves. The data can be used by academics, students, and researchers working on development of anticancer drugs.

**Table:** Specifications Table

Subject	*Biochemistry, Nanomedicine*
Specific subject area	*Cancer Research*
Type of data	Tables Graphs
How data were acquired	Cell culture (DMEM and RPMI 1640 used as growth media), Inverted microscope (Olympus), microplate reader (Infinite M1000, Tecan)
Data format	Raw Analyzed
Parameters for data collection	Cells were maintained in appropriate growth media in an incubator at 37 °C, 5% CO_2_; and 95% humidity. HeLa Cells were grown in DMEM while PC3 and PNT1A were grown in RPMI 1640. Cells passaged 1–2 times a week. Cells were harvested and assayed at 60–75% confluence.
Description of data collection	The cytotoxicity was determined by measuring the absorbance signal of resazurin dye on the treated cell lines at 570nm and 600nm in a microplate reader.
Data source location	Institution: United States Army Medical Research Directorate - Kenya (USAMRD-K), Department of Emerging Infectious Diseases (DEID), Influenza Laboratory City: Nairobi Country: Kenya
Data accessibility	With the article

**Table:** 

**Value of the Data** • The data shows potential anticancer activity of AgNPs on HeLa, PC3 and PNT1A cells and highlights differences in cytotoxic activity of the treatments on the cell lines. • The data can be used by academics, students, researchers and industrialists poised in the cancer drugs development. • The data can be used to elucidate the 50% inhibitory concentration (IC_50_) values of the treatments on the cancer cell lines. • The data can be used to investigate the selectivity of the treatments by elucidating the selectivity indices, and thus inform researchers about the safety of such treatments. • The data can be highlighted for further studies in development of better anticancer drugs using green synthesized nanoparticles.

## 1 Data

**Fig. 1:**
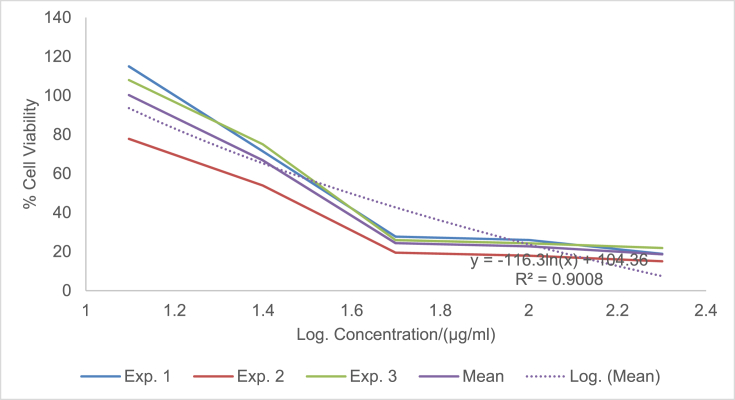
A graph showing the Cytotoxicity of AgNPs-F against HeLa Cells using the Resazurin Metabolic Assay.

**Fig. 2:**
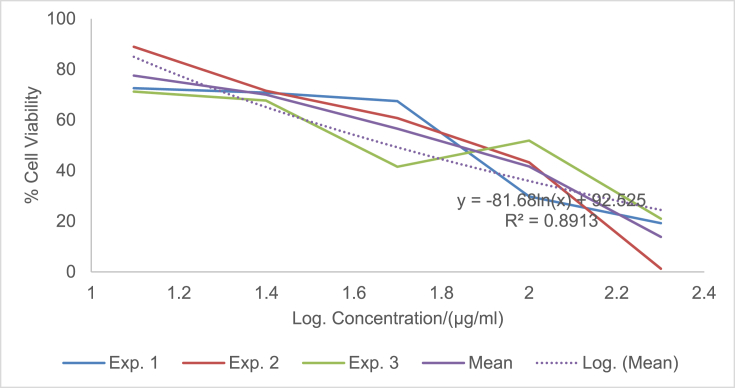
A graph showing the Cytotoxicity of AgNPs-F against PC3 Cells using the Resazurin Metabolic Assay.

**Fig. 3:**
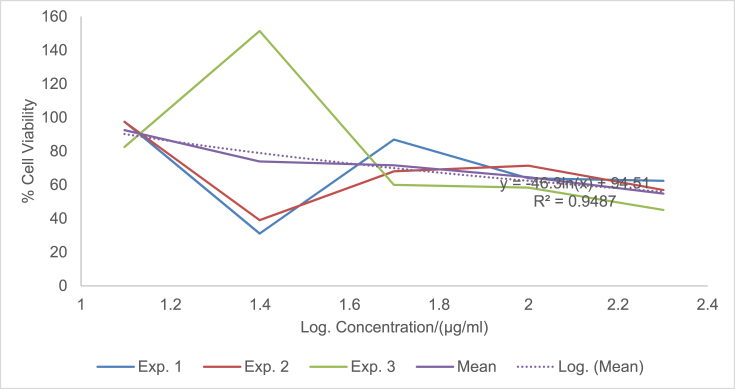
A graph showing the Cytotoxicity of AgNPs-F against PNT1A Cells using the Resazurin Metabolic Assay.

**Fig. 4:**
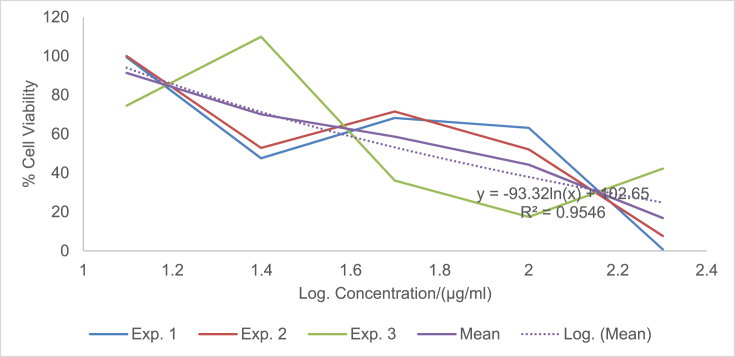
A graph showing the Cytotoxicity of AgNPs-L against HeLa Cells using the Resazurin Metabolic Assay.

**Fig. 5:**
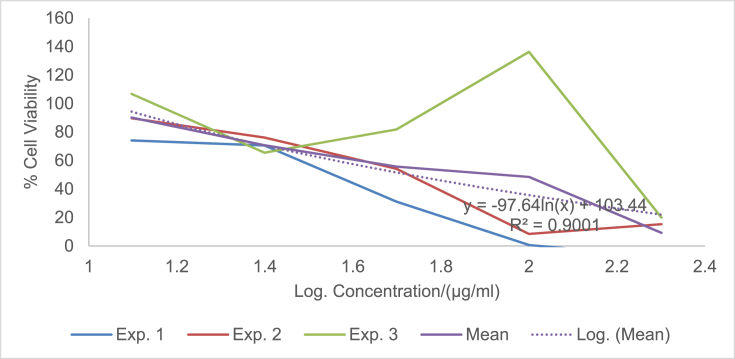
A graph showing the Cytotoxicity of AgNPs-L against PC3 Cells using the Resazurin Metabolic Assay.

**Fig. 6:**
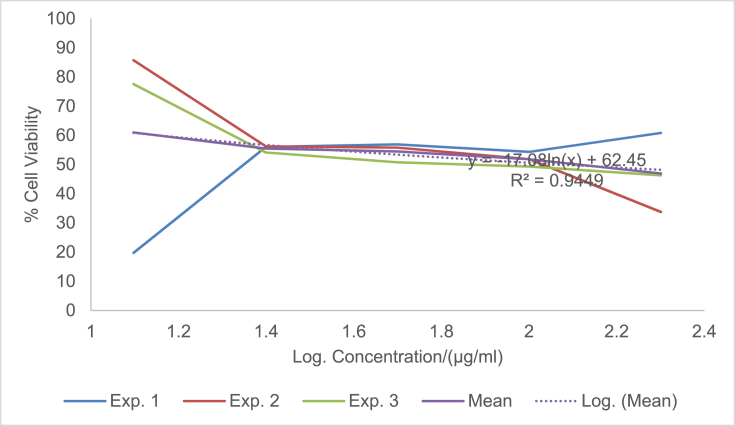
A graph showing the Cytotoxicity of AgNPs-L against PNT1A Cells using the Resazurin Metabolic Assay.

**Fig. 7:**
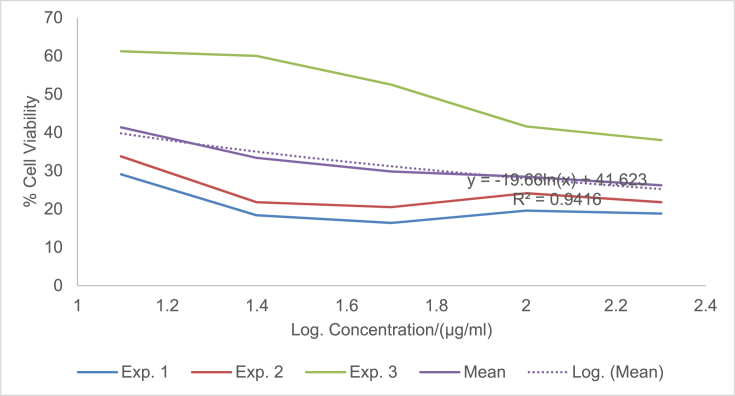
A graph showing the Cytotoxicity of 5FU against HeLa Cells using the Resazurin Assay.

**Fig. 8:**
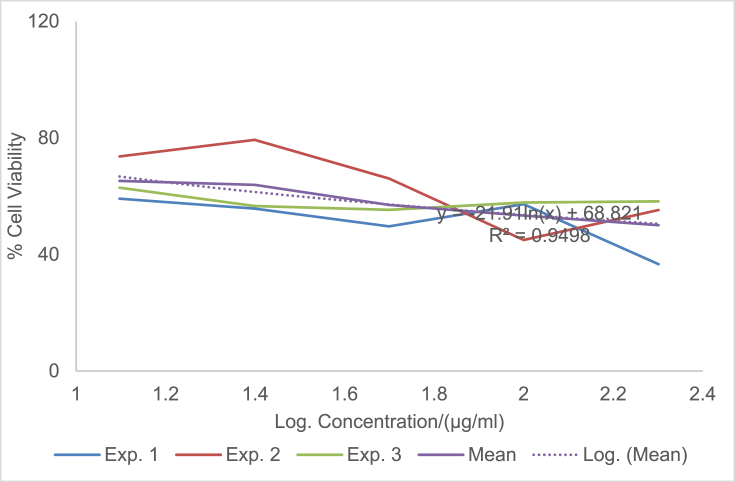
A graph showing the Cytotoxicity of 5FU against PC3 Cells using the Resazurin Metabolic Assay.

**Fig. 9:**
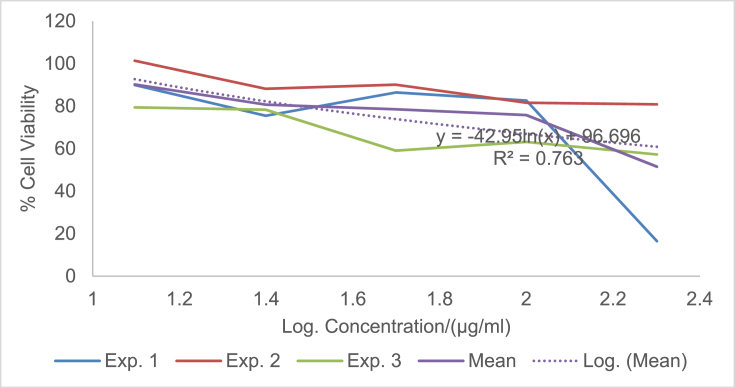
A graph showing the Cytotoxicity of 5FU against PNT1A Cells using the Resazurin Metabolic Assay.

The raw data of the treatments against the HeLa, PC3 and PNT1A cells are shown in the different Tables described in this section. Each experiment (Exp.) was represented by four independent replicates (Rep.). Table 1, Table 2, Table 3 show the data for cytotoxicity of silver nanoparticles derived from fruits extracts of *Annona muricata* (AgNPs-F) on HeLa, PC3 and PNT1A cells; Table 4, Table 5, Table 6 show the data for cytotoxicity of silver nanoparticles derived from leaves extracts of *Annona muricata* (AgNPs-L) on HeLa, PC3 and PNT1A cells; while Table 7, Table 8, Table 9 show the data for cytotoxicity of 5FU on HeLa, PC3 and PNT1A cells. On the other hand, Fig. 1, Fig. 2, Fig. 3 show the growth inhibition curves for cells treated with AgNPs-F, Fig. 4, Fig. 5, Fig. 6 show growth inhibition curves of cells treated with AgNPs-L, while Fig. 7, Fig. 8, Fig. 9 show the growth inhibition curves for cells treated with 5FU.

**Table 1:** Data for cytotoxicity of the AgNPs-F against HeLa Cells using the Resazurin metabolic assay.

	Concentration (μg/ml)							
	Wavelength/nm	Replicates	Blank	200	100	50	25	12.5
Exp. 1	A570	Rep. 1	1.4698	1.3135	1.2295	1.2667	1.3932	1.4757
		Rep. 2	1.4291	0.9098	1.2994	1.2886	1.3688	1.4914
		Rep. 3	1.5494	1.3617	1.2275	1.3184	1.3802	1.4947
		Rep. 4	1.3866	0.8855	1.3176	1.2926	1.3883	1.5741
	Mean Absorbance of Reps.		1.45873	1.11763	1.2685	1.29158	1.38263	1.50897
	A600	Rep. 1	0.775	1.1489	1.0559	1.0849	0.8845	0.646
		Rep. 2	0.8197	0.8186	1.1004	1.0854	0.9019	0.8163
		Rep. 3	0.6554	1.1691	1.0688	1.1144	0.8115	0.6728
		Rep. 4	0.7578	0.7987	1.1132	1.0988	0.9122	0.636
	Mean Absorbance of Reps.		0.75197	0.98383	1.08457	1.09587	0.87753	0.69277
	Net Absorbance (A570-A600)		0.70676	0.1338	0.18393	0.19571	0.5051	0.8162
	% Cell Viability		100	18.9315	26.0244	27.6911	71.4670	115
Exp. 2	A570	Rep. 1	1.5427	1.2109	0.9767	1.3123	1.323	1.444
		Rep. 2	1.5653	0.927	1.1317	1.1178	1.3343	1.5014
		Rep. 3	1.4661	1.2944	1.2287	1.4169	1.3551	1.4673
		Rep. 4	1.4413	0.9868	1.1181	1.0673	1.4321	1.4468
	Mean Absorbance of Reps.		1.50385	1.10477	1.1138	1.22857	1.36113	1.46488
	A600	Rep. 1	0.5542	1.0557	0.8384	1.1167	0.7809	0.6737
		Rep. 2	0.4877	0.8098	0.961	0.9482	0.8498	0.5827
		Rep. 3	0.4531	1.0748	1.0026	1.1551	0.8375	0.6709
		Rep. 4	0.4348	0.8631	0.9213	0.8983	0.7712	0.7523
	Mean Absorbance of Reps.		0.48245	0.95085	0.93082	1.02958	0.80985	0.6699
	Net Absorbance (A570-A600)		1.0214	0.15392	0.18298	0.19899	0.55125	0.79498
	% Cell Viability		100	15.0695	17.9146	19.4821	53.9729	77.8324
Exp. 3	A570	Rep. 1	1.446	1.313	1.2562	1.2606	1.4306	1.4939
		Rep. 2	1.4997	1.1884	1.3107	1.4239	1.4366	1.5769
		Rep. 3	1.3913	1.1504	1.2825	1.534	1.4756	1.5519
		Rep. 4	1.3499	1.2011	1.3006	1.2713	1.441	1.6484
	Mean Absorbance of Reps.		1.42172	1.21323	1.2875	1.37245	1.44595	1.56777
	A600	Rep. 1	0.503	1.091	1.0543	1.061	0.6198	0.4778
		Rep. 2	0.3938	0.9762	1.0516	1.1493	0.7063	0.5043
		Rep. 3	0.3751	0.9297	1.0299	1.2124	0.6676	0.4227
		Rep. 4	0.4032	0.9783	1.0403	1.027	0.778	0.5372
	Mean Absorbance of Reps.		0.41877	0.9938	1.04402	1.11242	0.69292	0.4855
	Net Absorbance (A570-A600)		1.00295	0.21943	0.24348	0.26003	0.75303	1.08227
	% Cell Viability		100	21.8788	24.2763	25.9265	75.0815	108
% Mean Cell Viability ± SD			100 ± 0	18.6266 ± 3.40	22.7384 ± 4.27	24.3666 ± 4.32	66.8405 ± 11.29	100.2775 ± 19.75

**Table 2:** Data for cytotoxicity of the AgNPs-F against PC3 Cells using the Resazurin metabolic assay.

	Concentration (μg/ml)							
	Wavelength/nm	Replicates	Blank	200	100	50	25	12.5
Exp. 1	A570	Rep. 1	1.3333	1.1585	1.0472	1.1885	1.2751	1.2804
		Rep. 2	1.2456	1.2892	1.1179	1.1709	1.2578	1.5277
		Rep. 3	1.2113	1.3057	1.0965	1.2815	1.3069	1.6487
		Rep. 4	1.1547	1.31	0.9138	1.1196	1.2775	1.6191
	Mean Absorbance of Reps.		1.23623	1.26585	1.04385	1.19013	1.27933	1.51897
	A600	Rep. 1	0.8743	1.0464	1.0163	0.9057	0.9813	0.9847
		Rep. 2	0.7974	1.1854	0.8347	0.8893	0.9626	1.234
		Rep. 3	0.8159	1.2455	0.8202	0.9755	0.9999	1.3307
		Rep. 4	0.7762	1.2624	1.003	0.8564	0.9837	1.3069
	Mean Absorbance of Reps.		0.81595	1.18492	0.91855	0.90673	0.98187	1.21408
	Net Absorbance (A570-A600)		0.42028	0.08092	0.1253	0.2834	0.29745	0.3049
	% Cell Viability		100	19.2552	29.8138	67.432	70.7751	72.5477
Exp. 2	A570	Rep. 1	1.1529	1.4277	1.0416	1.1698	1.2876	1.6169
		Rep. 2	1.302	1.1672	1.1424	1.1884	1.344	1.7367
		Rep. 3	1.2233	1.2382	1.8403	1.1754	1.2491	1.4129
		Rep. 4	1.1472	1.1836	1.0647	1.2895	1.34	1.4508
	Mean Absorbance of Reps.		1.20635	1.25417	1.27225	1.20578	1.30518	1.55432
	A600	Rep. 1	0.6568	1.7462	0.7479	0.9004	0.9852	1.2206
		Rep. 2	0.8626	1.0341	0.8102	0.9322	1.0424	1.2958
		Rep. 3	0.8162	1.2045	1.7465	0.9557	0.9652	1.0876
		Rep. 4	0.8145	1.0109	1.0593	1.0168	1.0288	1.1233
	Mean Absorbance of Reps.		0.78752	1.24893	1.09097	0.95128	1.0054	1.18182
	Net Absorbance (A570-A600)		0.41883	0.00525	0.18128	0.2545	0.29978	0.3725
	% Cell Viability		100	1.2535	43.2818	60.7652	71.5752	88.9393
Exp. 3	A570	Rep. 1	1.1465	1.1826	1.2776	1.3187	1.4139	1.3651
		Rep. 2	1.1237	1.2939	1.2806	1.0512	1.374	1.6522
		Rep. 3	1.3081	1.2568	1.2649	1.401	1.3359	1.2984
		Rep. 4	1.053	1.228	1.111	1.2029	1.4066	1.7333
	Mean Absorbance of Reps.		1.15782	1.24033	1.23352	1.24345	1.3826	1.51225
	A600	Rep. 1	0.54	1.0571	0.9571	1.1027	1.1306	1.0439
		Rep. 2	0.6766	1.2622	0.9634	1.0164	1.0716	1.3143
		Rep. 3	0.928	1.1568	1.0648	1.1281	1.0312	1.0449
		Rep. 4	0.7152	1.1127	1.0298	0.9907	1.0983	1.3841
	Mean Absorbance of Reps.		0.71495	1.1472	1.00378	1.05948	1.08292	1.1968
	Net Absorbance (A570-A600)		0.44287	0.09313	0.22975	0.18398	0.29967	0.31545
	% Cell Viability		100	21.0274	51.8769	41.5411	67.6658	71.2278
% Mean Cell Viability ± SD			100 ± 0	13.8454 ± 10.94	41.6575 ±11.12	56.5795 ± 13.44	70.0054 ± 2.07	77.5716 ± 9.87

**Table 3:** Data for cytotoxicity of the AgNPs-F against PNT1A normal cells using the Resazurin metabolic assay.

	Concentration (μg/ml)							
	Wavelength/nm	Replicates	Blank	200	100	50	25	12.5
Exp. 1	A570	Rep. 1	1.0662	1.322	1.1746	1.3105	0.9654	1.0308
		Rep. 2	1.0252	1.2622	1.1472	1.1819	1.1266	1.146
		Rep. 3	1.0735	1.5141	1.1129	1.0966	1.2235	2.0434
		Rep. 4	1.0679	1.4703	1.0964	1.2052	1.2261	1.1985
	Mean Absorbance of Reps.		1.0582	1.39215	1.13278	1.19855	1.1354	1.35468
	A600	Rep. 1	0.4355	0.9898	0.7002	0.6334	0.775	0.669
		Rep. 2	0.5535	0.9617	0.7756	0.733	0.9649	0.5
		Rep. 3	0.507	1.1626	0.7718	0.6998	1.1027	1.3381
		Rep. 4	0.5895	1.1143	0.9109	0.8611	1.0304	0.8164
	Mean Absorbance of Reps.		0.52138	1.0571	0.78963	0.73183	0.96825	0.83087
	Net Absorbance (A570-A600)		0.53682	0.33505	0.34315	0.46673	0.16715	0.5238
	% Cell Viability		100	62.4133	63.9221	86.9417	31.1368	97.5737
Exp. 2	A570	Rep. 1	1.0403	1.0461	1.108	1.1146	1.0012	1.053
		Rep. 2	1.0948	1.2727	1.0925	1.105	1.178	1.1035
		Rep. 3	1.0239	1.1515	1.0847	1.1341	1.2848	1.1462
		Rep. 4	1.0595	1.3475	1.0336	1.0617	1.2899	1.0691
	Mean Absorbance of Reps.		1.05463	1.20445	1.0797	1.10385	1.18847	1.09295
	A600	Rep. 1	0.6439	0.8089	0.6201	0.6013	0.7937	0.483
		Rep. 2	0.456	0.9612	0.8254	0.7689	0.9855	0.5061
		Rep. 3	0.6273	0.8999	0.6621	0.8289	1.1305	0.6187
		Rep. 4	0.4868	1.0054	0.7801	0.8518	1.0603	0.8104
	Mean Absorbance of Reps.		0.5535	0.91885	0.72193	0.76272	0.9925	0.60455
	Net Absorbance (A570-A600)		0.50113	0.2856	0.35777	0.34112	0.19598	0.4884
	% Cell Viability		100	56.9918	71.3944	68.0718	39.107	97.4607
Exp. 3	A570	Rep. 1	1.4067	1.4349	1.1762	1.2242	1.7042	1.2268
		Rep. 2	1.3437	1.3423	1.2115	1.2436	1.6892	1.3645
		Rep. 3	1.1885	1.3744	1.1532	1.2279	1.5748	1.3597
		Rep. 4	1.3056	1.3265	1.1547	1.3041	1.7545	1.3102
	Mean Absorbance of Reps.		1.31113	1.36953	1.1739	1.24995	1.68068	1.3153
	A600	Rep. 1	0.8095	1.128	0.8524	0.8504	0.7139	0.9781
		Rep. 2	0.5971	1.0511	0.7117	0.7322	0.6383	0.7012
		Rep. 3	0.5568	1.0699	0.8382	0.9259	0.7067	0.6284
		Rep. 4	0.6509	1.0417	0.7584	0.9122	0.6807	0.7825
	Mean Absorbance of Reps.		0.65358	1.07268	0.79018	0.85517	0.6849	0.77255
	Net Absorbance (A570-A600)		0.65755	0.29685	0.38373	0.39478	0.99578	0.54275
	% Cell Viability		100	45.1449	58.3568	60.0373	151.437	82.5412
% Mean Cell Viability ± SD			100 ± 0	54.85 ±8.83	64.5578 ± 6.54	71.6836 ± 13.81	73.8936 ± 67.27	92.5252 ± 8.65

**Table 4:** Data for cytotoxicity of the AgNPs-L against HeLa Cells using the Resazurin metabolic assay.

	Concentration (μg/ml)							
	Wavelength/nm	Replicates	Blank	200	100	50	25	12.5
Exp. 1	A570	Rep. 1	0.9300	0.8548	1.0364	0.9733	0.9654	0.9224
		Rep. 2	1.3646	1.0182	1.2764	0.9654	1.1266	1.1541
		Rep. 3	1.2094	1.3914	1.3338	1.2995	1.2235	1.1968
		Rep. 4	1.3059	1.1796	1.2899	1.1952	1.2261	1.2324
	Mean Absorbance of Reps.		1.20247	1.111	1.23412	1.10835	1.1354	1.12642
	A600	Rep. 1	0.6142	0.8395	0.8456	0.7411	0.775	0.5721
		Rep. 2	0.9294	0.9751	1.0388	0.6971	0.9649	0.6962
		Rep. 3	1.0003	1.4323	1.0919	1.1155	1.1027	0.9274
		Rep. 4	0.8611	1.1857	1.0725	0.9207	1.0304	0.9128
	Mean Absorbance of Reps.		0.85125	1.10815	1.0122	0.8686	0.96825	0.77713
	Net Absorbance (A570-A600)		0.35122	0.00285	0.22192	0.23975	0.16715	0.3493
	% Cell Viability		100	0.81144	63.186	68.2611	47.5906	99.4519
Exp. 2	A570	Rep. 1	0.9551	0.8854	1.0429	1.0087	1.0012	0.9341
		Rep. 2	1.4089	1.0653	1.2939	1.006	1.178	1.1937
		Rep. 3	1.2444	1.4537	1.362	1.3543	1.2848	1.2442
		Rep. 4	1.3292	1.2382	1.3149	1.2499	1.2899	1.264
	Mean Absorbance of Reps.		1.2344	1.16065	1.25343	1.15473	1.18847	1.159
	A600	Rep. 1	0.6179	0.8533	0.8723	0.7601	0.7937	0.5722
		Rep. 2	0.9504	0.9948	1.0855	0.7129	0.9855	0.712
		Rep. 3	1.0168	1.4649	1.1522	1.1398	1.1305	0.9507
		Rep. 4	0.8705	1.2156	1.1315	0.945	1.0603	0.9185
	Mean Absorbance of Reps.		0.8639	1.13215	1.06038	0.88945	0.9925	0.78835
	Net Absorbance (A570-A600)		0.3705	0.0285	0.19305	0.26528	0.19598	0.37065
	% Cell Viability		100	7.6923	52.1053	71.5992	52.8947	100.04
Exp. 3	A570	Rep. 1	1.618	1.4486	1.4134	1.4438	1.7042	1.5772
		Rep. 2	1.6132	1.3799	1.3523	1.3666	1.6892	1.5764
		Rep. 3	1.5871	1.3893	1.3497	1.3177	1.5748	1.4538
		Rep. 4	2.2674	1.3500	1.3508	1.5011	1.7545	1.4657
	Mean Absorbance of Reps.		1.77143	1.39195	1.36655	1.4073	1.68068	1.51828
	A600	Rep. 1	0.7961	1.0479	1.2882	1.1222	0.7139	0.7698
		Rep. 2	0.8412	1.0662	1.1898	1.0908	0.6383	0.8527
		Rep. 3	0.8130	0.9942	1.1778	1.1227	0.7067	0.8380
		Rep. 4	1.0122	0.9290	1.1759	0.9830	0.6807	0.9100
	Mean Absorbance of Reps.		0.86563	1.00932	1.20793	1.07968	0.6849	0.84263
	Net Absorbance (A570-A600)		0.9058	0.38263	0.15863	0.32762	0.99578	0.67565
	% Cell Viability		100	42.2417	17.5121	36.1697	109.933	74.5915
% Mean Cell Viability ± SD			100 ± 0	16.9151 ± 22.2	44.2678 ± 23.82	58.6767 ± 19.56	70.1395 ± 34.56	91.3613 ± 14.53

**Table 5:** Data for cytotoxicity of the AgNPs-L against PC3 Cells using the Resazurin metabolic assay.

	Concentration (μg/ml)								
	Wavelength/nm	Replicates		Blank	200	100	50	25	12.5
Exp. 1	A570	Rep. 1		1.0656	1.0033	0.8548	1.0472	1.1885	1.2751
		Rep. 2		1.0504	0.8598	1.0182	1.1179	1.1709	1.2578
		Rep. 3		0.9836	1.1382	1.3914	1.0965	1.2815	1.3069
		Rep. 4		0.9948	0.9712	1.1796	0.9138	1.1196	1.2775
	Mean Absorbance of Reps.			1.0236	0.99312	1.111	1.04385	1.19013	1.27933
	A600		Rep. 1	0.6045	1.0854	0.8395	1.0163	0.9057	0.9813
			Rep. 2	0.6152	0.8481	0.9751	0.8347	0.8893	0.9626
			Rep. 3	0.6472	1.1427	1.4323	0.8202	0.9755	0.9999
			Rep. 4	0.6218	1.0194	1.1857	1.003	0.8564	0.9837
	Mean Absorbance of Reps.			0.62217	1.0239	1.10815	0.91855	0.90673	0.98187
	Net Absorbance (A570-A600)			0.40143	−0.0307	0.00285	0.1253	0.2834	0.29745
	% Cell Viability			100	−7.6664	0.70997	31.2138	70.5985	74.0985
Exp. 2	A570	Rep. 1		0.9578	0.9334	0.8854	1.0416	1.1698	1.2876
		Rep. 2		1.1571	1.0754	1.0653	1.1424	1.1884	1.344
		Rep. 3		1.1137	1.0846	1.4537	1.8403	1.1754	1.2491
		Rep. 4		1.0617	0.9629	1.2382	1.0647	1.2895	1.34
	Mean Absorbance of Reps.			1.07257	1.01407	1.16065	1.27225	1.20578	1.30518
	A600	Rep. 1		0.5302	0.9295	0.8533	0.7479	0.9004	0.9852
		Rep. 2		0.8297	0.8506	0.9948	0.8102	0.9322	1.0424
		Rep. 3		0.764	1.1326	1.4649	1.7465	0.9557	0.9652
		Rep. 4		0.83	0.9381	1.2156	1.0593	1.0168	1.0288
	Mean Absorbance of Reps.			0.73847	0.9627	1.13215	1.09097	0.95128	1.0054
	Net Absorbance (A570-A600)			0.3341	0.05138	0.0285	0.18128	0.2545	0.29978
	% Cell Viability			100	15.3771	8.53037	54.2577	76.1748	89.7261
Exp. 3	A570	Rep. 1		1.323	1.132	1.4486	1.2776	1.3187	1.4139
		Rep. 2		1.2966	1.2185	1.3799	1.2806	1.0512	1.374
		Rep. 3		1.2871	1.1995	1.3893	1.2649	1.401	1.3359
		Rep. 4		1.209	1.1804	1.35	1.111	1.2029	1.4066
	Mean Absorbance of Reps.			1.27892	1.1826	1.39195	1.23352	1.24345	1.3826
	A600	Rep. 1		1.0241	1.0888	1.0479	0.9571	1.1027	1.1306
		Rep. 2		0.9343	1.0871	1.0662	0.9634	1.0164	1.0716
		Rep. 3		0.9466	1.1122	0.9942	1.0648	1.1281	1.0312
		Rep. 4		1.0884	1.2177	0.929	1.0298	0.9907	1.0983
	Mean Absorbance of Reps.			0.99835	1.12645	1.00932	1.00378	1.05948	1.08292
	Net Absorbance (A570-A600)			0.28057	0.05615	0.38263	0.22975	0.18398	0.29967
	% Cell Viability			100	20.0125	136.372	81.8854	65.5707	106.807
% Mean Cell Viability ± SD				100 ± 0	9.24106 ± 7.16	48.5374 ± 76.16	55.7856 ± 25.37	70.7813 ± 5.3	90.2107 ± 16.36

**Table 6:** Data for cytotoxicity of the AgNPs-L against PNT1A normal cells using the Resazurin metabolic assay.

	Concentration (μg/ml)							
	Wavelength/nm	Replicates	Blank	200	100	50	25	12.5
Exp. 1	A570	Rep. 1	1.2407	1.0866	1.2406	1.3137	1.2693	0.9282
		Rep. 2	1.1232	0.8972	1.28	1.2804	1.2856	0.9183
		Rep. 3	1.269	1.5388	1.1828	1.2567	1.069	0.9439
		Rep. 4	1.2601	1.2797	1.2009	1.303	1.235	0.8617
	Mean Absorbance of Reps.		1.22325	1.20057	1.22607	1.28845	1.21472	0.91302
	A600	Rep. 1	0.7743	0.746	0.9653	1.0537	0.9954	0.4771
		Rep. 2	0.6654	0.9776	1.0465	1.0191	1.0113	1.0198
		Rep. 3	0.7117	0.95	0.9685	1.0086	0.8496	0.7099
		Rep. 4	0.9448	1.0352	0.9474	1.0497	0.9955	1.0908
	Mean Absorbance of Reps.		0.77405	0.9272	0.98192	1.03277	0.96295	0.8244
	Net Absorbance (A570-A600)		0.4492	0.27338	0.24415	0.25568	0.25177	0.08862
	% Cell Viability		100	60.8582	54.3522	56.9179	56.0496	19.7295
Exp. 2	A570	Rep. 1	1.1515	1.1619	1.1257	1.1935	1.1548	0.9842
		Rep. 2	1.211	1.3064	1.2002	1.1932	1.1526	1.3729
		Rep. 3	1.0892	1.382	1.4051	1.2978	1.2415	1.2624
		Rep. 4	1.2657	1.1699	1.1682	1.3704	1.2996	1.2985
	Mean Absorbance of Reps.		1.17935	1.25505	1.2248	1.26372	1.21213	1.2295
	A600	Rep. 1	0.5864	0.9968	0.8734	0.9459	0.9092	0.4771
		Rep. 2	0.7193	0.9433	0.9986	0.9383	0.8934	1.0198
		Rep. 3	0.71	1.2182	1.1052	1.0181	0.9764	0.7099
		Rep. 4	0.8115	1.2238	0.9416	1.0979	1.0063	1.0908
	Mean Absorbance of Reps.		0.7068	1.09552	0.9797	1.00005	0.94633	0.8244
	Net Absorbance (A570-A600)		0.47255	0.15952	0.2451	0.26367	0.2658	0.4051
	% Cell Viability		100	33.7583	51.8675	55.7983	56.248	85.7264
Exp. 3	A570	Rep. 1	1.1275	1.035	1.2563	1.3947	1.2913	1.1149
		Rep. 2	1.2802	1.156	1.1997	1.4059	1.3251	1.246
		Rep. 3	1.2155	1.1991	1.2531	1.0914	1.3846	1.2462
		Rep. 4	1.2674	1.2178	1.2982	1.4632	1.3368	1.2599
	Mean Absorbance of Reps.		1.22265	1.15198	1.25183	1.3388	1.33445	1.21675
	A600	Rep. 1	0.8274	0.746	0.9999	1.1287	1.0471	0.9102
		Rep. 2	0.6811	0.9776	0.979	1.1563	1.0674	0.623
		Rep. 3	0.6737	0.95	1.0378	0.8926	1.0979	0.9252
		Rep. 4	0.7668	1.0352	1.0329	1.1917	1.0738	0.9023
	Mean Absorbance of Reps.		0.73725	0.9272	1.0124	1.09232	1.07155	0.84018
	Net Absorbance (A570-A600)		0.4854	0.22478	0.23943	0.24648	0.2629	0.37657
	% Cell Viability		100	46.3072	49.3253	50.7777	54.1615	77.5803
% Mean Cell Viability ± SDs			100 ± 0	46.9746 ± 13.56	51.8483 ± 2.51	54.498 ± 3.23	55.4864 ± 1.15	61.0121 ± 35.98

**Table 7:** Data for cytotoxicity of 5FU against HeLa Cells using the Resazurin metabolic assay.

	Concentration (μg/ml)							
	Wavelength/nm	Replicates	Blank	200	100	50	25	12.5
Exp. 1	A570	Rep. 1	0.9300	0.9917	1.2199	0.8006	1.0193	1.0169
		Rep. 2	1.3646	1.186	1.2638	1.1729	1.1181	1.207
		Rep. 3	1.2094	1.0287	1.1894	1.1752	1.0646	1.1992
		Rep. 4	1.3059	1.171	1.1563	1.0809	1.1307	1.1797
	Mean Absorbance of Reps.		1.20247	1.09435	1.20735	1.0574	1.08318	1.1507
	A600	Rep. 1	0.6142	0.8624	1.127	0.699	0.9253	0.8917
		Rep. 2	0.9294	1.1043	1.1901	1.0642	1.074	1.1148
		Rep. 3	1.0003	1.0022	1.1349	1.1609	1.0259	1.0741
		Rep. 4	0.8611	1.144	1.102	1.0754	1.0492	1.1133
	Mean Absorbance of Reps.		0.85125	1.02823	1.1385	0.99988	1.0186	1.04847
	Net Absorbance (A570-A600)		0.35122	0.06612	0.06885	0.05752	0.06457	0.10223
	% Cell Viability		100	18.827	19.6028	16.3784	18.3856	29.1053
Exp. 2	A570	Rep. 1	0.9551	1.017	1.2367	0.8372	1.0507	1.0592
		Rep. 2	1.4089	1.2117	1.3115	1.2111	1.1562	1.2348
		Rep. 3	1.2444	1.0983	1.2719	1.2354	1.12	1.2559
		Rep. 4	1.3292	1.2225	1.213	1.1333	1.1685	1.2467
	Mean Absorbance of Reps.		1.2344	1.13737	1.25828	1.10425	1.12385	1.19915
	A600	Rep. 1	0.6179	0.8865	1.1511	0.7214	0.9486	0.9195
		Rep. 2	0.9504	1.1268	1.2173	1.0868	1.0954	1.1367
		Rep. 3	1.0168	1.0383	1.1735	1.2021	1.0573	1.1011
		Rep. 4	0.8705	1.1749	1.1333	1.1027	1.0712	1.1387
	Mean Absorbance of Reps.		0.8639	1.05663	1.1688	1.02825	1.04312	1.074
	Net Absorbance (A570-A600)		0.3705	0.08075	0.08948	0.076	0.08072	0.12515
	% Cell Viability		100	21.7949	24.1498	20.5128	21.7881	33.7787
Exp. 3	A570	Rep. 1	1.618	1.3869	1.4109	1.4461	1.5886	1.51
		Rep. 2	1.6132	1.2718	1.4172	1.5096	1.4624	1.5024
		Rep. 3	1.5871	1.306	1.4215	1.5138	1.374	1.4649
		Rep. 4	2.2674	1.2012	1.393	1.3669	1.3833	1.4478
	Mean Absorbance of Reps.		1.77143	1.29147	1.41065	1.4591	1.45207	1.48128
	A600	Rep. 1	0.7961	0.9932	0.9462	1.0964	0.7275	0.9649
		Rep. 2	0.8412	0.8292	1.0772	1.0013	0.9073	0.8436
		Rep. 3	0.813	0.9733	1.0002	0.8611	1.0423	0.9563
		Rep. 4	1.0122	0.9924	1.1114	0.9739	0.9566	0.9414
	Mean Absorbance of Reps.		0.86563	0.94703	1.03375	0.98318	0.90843	0.92655
	Net Absorbance (A570-A600)		0.9058	0.34445	0.3769	0.47592	0.54365	0.55473
	% Cell Viability		100	38.0272	41.6096	52.542	60.0188	61.2414
Mean % Cell Viability ± SD			100 ± 0	26.2163 ± 10.33	28.4541 ± 11.62	29.811 ± 19.79	33.3975 ± 23.11	41.3751 ± 17.36

**Table 8:** Data for cytotoxicity of 5FU against PC3 Cells using the Resazurin metabolic assay.

	Concentration (μg/ml)							
	Wavelength/nm	Replicates	Blank	200	100	50	25	12.5
Exp. 1	A570	Rep. 1	1.3333	1.2295	1.4598	1.3509	1.5099	1.3809
		Rep. 2	1.2456	1.2565	1.3126	1.2195	1.3586	1.259
		Rep. 3	1.2113	1.2528	1.4194	1.2509	1.454	1.277
		Rep. 4	1.1547	1.219	1.3856	1.2766	1.3622	1.2752
	Mean Absorbance of Reps.		1.23623	1.23945	1.39435	1.27448	1.42118	1.29802
	A600	Rep. 1	0.8743	1.1243	1.3535	1.0984	1.3006	1.1187
		Rep. 2	0.7974	1.058	1.1941	1.0904	1.1127	0.9825
		Rep. 3	0.8159	1.101	0.969	0.9938	1.2125	1.0203
		Rep. 4	0.7762	1.0578	1.0978	1.0803	1.1213	1.0756
	Mean Absorbance of Reps.		0.81595	1.08527	1.1536	1.06572	1.18677	1.04928
	Net Absorbance (A570-A600)		0.42028	0.15418	0.24075	0.20875	0.2344	0.24875
	% Cell Viability		100	36.6843	57.2839	49.6699	55.773	59.1874
Exp. 2	A570	Rep. 1	1.1529	1.1256	1.0922	1.189	1.1088	1.2155
		Rep. 2	1.302	1.0736	2.0665	1.1893	1.2698	1.1701
		Rep. 3	1.2233	1.0479	1.2537	1.1769	1.2981	1.1123
		Rep. 4	1.1472	1.0659	1.0555	1.1281	1.1668	1.1998
	Mean Absorbance of Reps.		1.20635	1.07825	1.36698	1.17083	1.21088	1.17443
	A600	Rep. 1	0.6568	0.8058	0.6521	0.8702	0.7814	0.8249
		Rep. 2	0.8626	0.8734	2.0876	0.8702	0.8966	0.8882
		Rep. 3	0.8162	0.7755	0.9804	1.0262	0.9785	0.7266
		Rep. 4	0.8145	0.9318	0.9941	0.8094	0.8577	1.0238
	Mean Absorbance of Reps.		0.78752	0.84663	1.17855	0.894	0.87855	0.86587
	Net Absorbance (A570-A600)		0.41883	0.23162	0.18843	0.27682	0.33232	0.30855
	% Cell Viability		100	55.3035	44.989	66.0956	79.347	73.6704
Exp. 3	A570	Rep. 1	1.1465	1.0529	0.9879	1.4099	1.0342	1.215
		Rep. 2	1.1237	1.1887	1.251	1.2178	1.274	1.2613
		Rep. 3	1.3081	1.3231	1.2296	1.2199	1.3973	1.3347
		Rep. 4	1.053	1.2915	1.3369	1.3105	1.3249	1.3175
	Mean Absorbance of Reps.		1.15782	1.21405	1.20135	1.28953	1.2576	1.28212
	A600	Rep. 1	0.54	0.7176	0.7193	1.195	0.7924	0.9112
		Rep. 2	0.6766	0.8633	1.1052	0.8249	1.0303	1.0017
		Rep. 3	0.928	1.2248	0.9405	1.1032	1.1282	1.0617
		Rep. 4	0.7152	1.019	1.0162	1.0546	1.0763	1.039
	Mean Absorbance of Reps.		0.71495	0.95618	0.9453	1.04442	1.0068	1.0034
	Net Absorbance (A570-A600)		0.44287	0.25787	0.25605	0.2451	0.2508	0.27872
	% Cell Viability		100	58.2275	57.8154	55.3429	56.63	62.9354
Mean % Cell Viability ± SD			100 ± 0	50.0718 ± 11.69	53.3628 ± 7.26	57.0361 ± 8.34	63.9167 ± 13.37	65.2644 ± 7.52

**Table 9:** Data for cytotoxicity of 5FU against PNT1A normal cells using the Resazurin metabolic assay.

	Concentration (μg/ml)							
	Wavelength/nm	Replicates	Blank	200	100	50	25	12.5
Exp. 1	A570	Rep. 1	1.0662	0.9282	1.0414	1.085	1.0312	1.0644
		Rep. 2	1.0252	0.9183	1.1152	1.0759	1.0472	1.1821
		Rep. 3	1.0735	0.9439	1.2742	1.1065	1.1835	1.2094
		Rep. 4	1.0679	0.8617	0.9168	1.1691	1.0481	1.1196
	Mean Absorbance of Reps.		1.0582	0.91302	1.0869	1.10913	1.0775	1.14388
	A600	Rep. 1	0.4355	0.4771	0.47	0.482	0.5063	0.5058
		Rep. 2	0.5535	1.0198	0.675	0.6988	0.6371	0.6727
		Rep. 3	0.507	0.7099	0.8579	0.6031	0.852	0.7457
		Rep. 4	0.5895	1.0908	0.5706	0.7968	0.6933	0.7201
	Mean Absorbance of Reps.		0.52138	0.8244	0.64338	0.64518	0.67217	0.66107
	Net Absorbance (A570-A600)		0.53682	0.08862	0.44352	0.46395	0.40533	0.4828
	% Cell Viability		100	16.5091	82.62	86.4248	75.5041	89.9362
Exp. 2	A570	Rep. 1	1.0403	0.9842	0.9956	1.0839	1.0614	1.0555
		Rep. 2	1.0948	1.3729	1.0657	1.088	0.9943	0.9717
		Rep. 3	1.0239	1.2624	0.9841	1.0846	1.0802	1.1183
		Rep. 4	1.0595	1.2985	1.0744	1.0832	1.07	1.1113
	Mean Absorbance of Reps.		1.05463	1.2295	1.02995	1.08492	1.05148	1.0642
	A600	Rep. 1	0.6439	0.4771	0.5945	0.4893	0.4823	0.4356
		Rep. 2	0.456	1.0198	0.5494	0.6673	0.6906	0.5797
		Rep. 3	0.6273	0.7099	0.701	0.6897	0.633	0.5618
		Rep. 4	0.4868	1.0908	0.6393	0.6875	0.6322	0.6472
	Mean Absorbance of Reps.		0.5535	0.8244	0.62105	0.63345	0.60953	0.55607
	Net Absorbance (A570-A600)		0.50113	0.4051	0.4089	0.45147	0.44195	0.50812
	% Cell Viability		100	80.8381	81.5964	90.0923	88.1916	101.397
Exp. 3	A570	Rep. 1	1.4067	1.1149	1.2754	1.2814	1.2513	1.3242
		Rep. 2	1.3437	1.246	1.1233	1.2189	1.2208	1.216
		Rep. 3	1.1885	1.2462	1.1105	1.1816	1.2943	1.1357
		Rep. 4	1.3056	1.2599	1.1501	1.0069	1.2266	1.1305
	Mean Absorbance of Reps.		1.31113	1.21675	1.16482	1.1722	1.24825	1.2016
	A600	Rep. 1	0.8095	0.9102	0.8707	1.0449	0.9541	0.7732
		Rep. 2	0.5971	0.623	0.6806	0.7104	0.6601	0.741
		Rep. 3	0.5568	0.9252	0.7461	0.708	0.6388	0.5442
		Rep. 4	0.6509	0.9023	0.7008	0.6725	0.6796	0.66
	Mean Absorbance of Reps.		0.65358	0.84018	0.74955	0.78395	0.73315	0.6796
	Net Absorbance (A570-A600)		0.65755	0.37657	0.41527	0.38825	0.5151	0.522
	% Cell Viability		100	57.2694	63.1549	59.0449	78.3362	79.3856
% Mean Cell Viability ± SD			100 ± 0	51.5389 ± 32.55	75.7904 ± 10.95	78.5207 ± 16.97	80.6773 ± 6.66	90.2396 ± 11.0

### 1.1 Data for cytotoxicity of AgNPs-F on HeLa, PC3 and PNT1A cells

.

### 1.2 Data for cytotoxicity of AgNPs-L on HeLa, PC3 and PNT1A cells

.

### 1.3 Data for cytotoxicity of 5FU on HeLa, PC3 and PNT1A cells

.

## 2 Experimental design, materials, and methods

### 2.1 Chemicals and reagents

All chemicals and reagents were procured from certified suppliers and were of the highest analytical standard. DMEM, RPMI1640, Penicillin/Streptomycin, Non-Essential Amino Acids, Trypsin-EDTA, and Resazurin were obtained from Solarbio (China). FCS, 5FU, Phosphate buffered saline (PBS) and Dimethyl Sulfoxide (DMSO) were obtained from Sigma Aldrich (Germany).

### 2.2 The silver nanoparticles

Previously prepared and characterized AgNPs from ethanolic extracts of fruits and leaves of *Annona muricata* were used for the study from which the current data was obtained [1], [2]. AgNPs-F used had an absorption maximum at 427 nm and were stable under different pH, Temperature and storage conditions. The AgNPs-F had an average crystalline size of 60.12 nm, a polydispersity index of 0.1235 and were spherical in nature. The functional groups responsible for the formation of the AgNPs included; Alkanes and alkyls, aldehydes and esters, nitro groups, alcohol groups, amines, amides, alkenes, acids and alkyl halides [1], [2]. On the other hand, AgNPs-L used had an absorption maximum at 429 nm and were stable under different pH, Temperature and storage conditions. The AgNPs-L had an average crystalline size of 87.36 nm, a polydispersity index of 0.16 and were spherical in nature. The functional groups responsible for the formation of the AgNPs included; Alkanes and alkyls, aldehydes and esters, nitro groups, alcohol groups, amines, amides, alkenes, acids and alkyl halides [1].

### 2.3 Cell lines

The HeLa and PC-3 cells were Cervical and Prostate adenocarcinomas respectively. On the other hand, the PNT1A cells were normal immortalized prostate cells. HeLa, PC3, and PNT1A were sourced from the European collection of Animal Cell Cultures (ECACC). All cells were adherent.

### 2.4 Cell culture

The cells were grown separately in appropriate media (HeLa in DMEM; PC3 and PNT1A in RPMI 1640) containing l-Glutamine and supplemented with 10% batch tested inactivated fetal calf serum (FCS), 1% Penicillin/Streptomycin, and 1% Non-essential amino acids. The cells were kept an incubator at 37 °C, 5% CO_2_; and 95% humidity. Cells were Trypsinized and passaged 1–2 times a week and were harvested and used for the assays during their logarithmic growth phase at about 60–75% confluence.

### 2.5 Preparation of the AgNPs solutions, 5-FU and blanks in media

AgNPs-F and AgNPs-L stock solutions (10mg/ml) were prepared by dispersing them in 0.5% DMSO in culture media. Briefly, 100mg of the AgNPs were dispersed in 10 mL of culture medium (containing Dimethyl Sulfoxide (DMSO) of 0.5%v/v). Required treatment concentrations of (200, 100, 50, 25, and 12.5 μg/mL were then made by dilutions of the stock solutions using the formula C_1_V_1_=C_2_V_2_. To prepare the standard anticancer treatment regimen of 5-FU, a stock solution was prepared as above and then diluted with culture media to desired concentrations ranging from 12.5 to 200 μg/mL. The final concentration of dimethyl sulfoxide (DMSO) in each cell culture did not exceed 1% v/v to keep the cytotoxicity of DMSO low [3].

### 2.6 Measurement of the anticancer activities of the AgNPs and 5FU using the Resazurin Assay

The effects of the AgNPs on each of the cell lines' viability and death was determined using the Resazurin (7-hydroxy-10-oxido- phenoxazin-10-ium-3-one) assay as previously described [4], [5], [6], [7]. Exponentially growing cells were harvested, washed and seeded in 96 well plates containing 0.5 × 10^4^Cells/well and incubated with 100 μL per well culture media and allowed to attach overnight. Seeding media was then removed from each of the plates. The attached cultured cells were then treated by adding of 100 μL of the treatments at concentrations of 200, 100, 50, 25, and 12.5μg/mL (in culture media). In addition, the DMSO alone in media was added to another set of cells as the solvent control blank (DMSO = 0.5%v/v). Standard drug 5-FU was used as a reference drug for cancer as positive control. The treated cells were then incubated in a humified CO_2_ incubator at 37 °C. 24 Hours from the start of the incubation, 20 μl resazurin at a concentration of 0.15mg/ml in PBS was added to each of the wells and then incubated at 37 °C for an additional 4 hours. After 4 hours from the addition of resazurin, the plates containing the treated cells were then retrieved from the incubator and the absorbance signal was quickly measured at 570/600nm (excitation/emission wavelengths), using a microplate reader (Infinite M1000, Tecan). Each treatment was read in at least four replicates.

### 2.7 How the data can be analyzed

The presented data can be analyzed by determining the percentage cell viability using the formula: % Viability = (Net absorbance of treated samples/Net absorbance of blank) ×100. The effect of the samples on the proliferation of the cell lines can then be expressed in form of graphs of percentage cell viability against logarithm of concentration as shown in Fig. 1, Fig. 2, Fig. 3, Fig. 4, Fig. 5, Fig. 6, Fig. 7, Fig. 8, Fig. 9 under the data section above. Fifty percent of inhibitory concentration (IC_50_) or cytotoxic concentration (CC_50_) of each of the treatments can then be calculated from the growth inhibition curves.

## Research clearance and registration

The study from which the current data was obtained was cleared by the PAUSTI board of examiners (MB400-0007/17), The Uganda National Council for Science and Technology (NS 43ES) as well as the Jomo Kenyatta University of Agriculture and Technology Institutional Ethics Review Committee (Ref. no: JKU/2/4/896B).
